# Heyde’s Syndrome: Presentation and Successful Treatment With Transcatheter Aortic Valve Implantation (TAVI)

**DOI:** 10.7759/cureus.94408

**Published:** 2025-10-12

**Authors:** Alexander Henshall, Robert Ambrogetti, Rodney De Palma

**Affiliations:** 1 Cardiology, Buckinghamshire Healthcare NHS Trust, Buckinghamshire, GBR

**Keywords:** echocardiogram, heyde's syndrome, myocardial infarction type 2, small bowel capsule endoscopy, upper gastrointestinal bleed

## Abstract

Heyde’s syndrome, characterized by the triad of severe aortic stenosis (AS), GI bleeding (GIB) from angiodysplasia, and acquired von Willebrand syndrome, presents a complex diagnostic and therapeutic challenge. We report the case of a male patient in his 70s with recurrent melena and profound anemia requiring multiple transfusions. Extensive initial investigations, including gastroscopy and colonoscopy, failed to identify a bleeding source. The diagnosis was suspected after echocardiography confirmed critical AS and was later confirmed by capsule endoscopy, which revealed bleeding small bowel angiodysplasia. Instead of pursuing direct endoscopic treatment of the angiodysplasia, the decision was made to address the underlying cause with transcatheter aortic valve implantation (TAVI). Following the procedure, the patient’s hemoglobin stabilized, and subsequent enteroscopy confirmed resolution of the angiodysplasia. This case highlights that in patients with AS and obscure GIB, maintaining a high index of suspicion for Heyde’s syndrome is crucial. It demonstrates that timely intervention with TAVI can serve as a definitive treatment by resolving the bleeding diathesis through correction of the shear-stress platelet insult and underscores the importance of a multidisciplinary approach between cardiology and gastroenterology to achieve optimal patient outcomes.

## Introduction

Upper GI bleeding (UGIB) is a common and potentially life-threatening presentation in emergency departments, frequently manifesting as hematemesis, melena, or a combination of both [1]. The initial clinical approach requires rapid resuscitation, risk stratification, and timely investigation, typically beginning with esophagogastroduodenoscopy (OGD) to identify common sources such as peptic ulcers or esophageal varices [1].

A less common but important etiology is Heyde’s syndrome, a clinical triad comprising severe aortic stenosis (AS), GI bleeding (GIB) from angiodysplasia, and acquired von Willebrand syndrome type 2A (AVWS-2A) [2]. The prevalence of AS increases significantly with age, affecting approximately 7.5% of the population over 75 years, with 1.8% having moderate or severe disease [3]. Although overt Heyde’s syndrome is rare, occurring in an estimated 1-3% of patients with significant AS, a subclinical coagulopathy is far more common [4]. The pathophysiological link is mechanical: high shear stress forces across the stenotic aortic valve cause proteolytic cleavage of von Willebrand factor (VWF) multimers, leading to an acquired deficiency that impairs platelet adhesion and promotes bleeding from susceptible vascular malformations (angiodysplasia) in the gut [4,5]. Importantly, this coagulopathy has been shown to reverse following successful aortic valve replacement (AVR), confirming the causal relationship [4].

This case report describes the management of a patient with recurrent GIB secondary to Heyde’s syndrome, highlighting the diagnostic challenges and the successful outcome achieved with transcatheter aortic valve implantation (TAVI). It underscores the importance of recognizing this syndrome to ensure timely diagnosis and appropriate management.

## Case presentation

A male patient in his 70s presented with melena and profound anemia, with an initial hemoglobin (Hb) level of 69 g/L. An OGD and CT of the chest, abdomen, and pelvis were performed but revealed no cause for the presenting melena and anemia. After transfusion of two units of red blood cells, the Hb stabilized, and the patient was discharged with an outpatient colonoscopy scheduled under the urgent “two-week wait” pathway for suspected cancer investigation. An ejection systolic murmur was also noted, and an outpatient echocardiogram was arranged. The patient’s medical history included hypercholesterolemia, type 2 diabetes mellitus, hypertension, and peripheral arterial disease, for which he had undergone a right femoral artery bypass graft 15 years earlier. Due to his peripheral arterial disease and previous bypass graft, he was taking clopidogrel, which was continued after the first admission.

**Figure 1:**
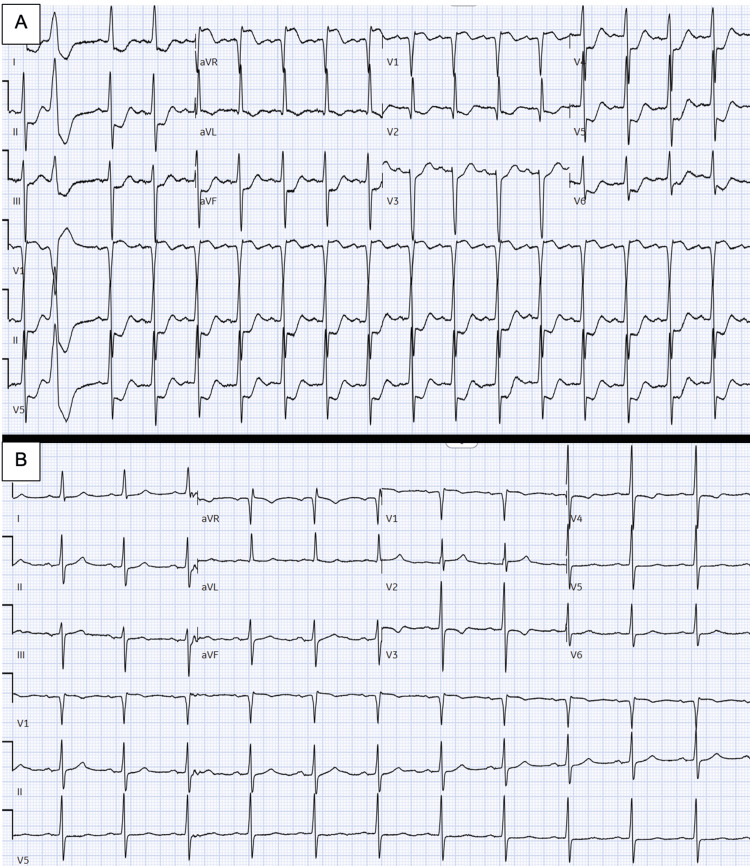
(A) ECG showing ischemic ST changes (widespread ST depression and ST elevation in leads aVR and V1) related to severe anemia. (B) ECG showing resolution of ischemic changes after blood transfusion.

Before the colonoscopy could be performed, 12 days later, the patient was admitted to cardiology with chest pain, ischemic ECG changes, dyspnea, and recurrent melena. Blood tests revealed more severe anemia, with Hb dropping to 54 g/L. The pronounced ischemic ECG changes included global ST depression and ST elevation in V1 and aVR (Figure 1). His initial high-sensitivity troponin I of 3,000 ng/L rose to a peak of >50,000 ng/L on serial measurements. Renal function remained stable throughout. In the setting of severe symptomatic anemia and melena, the presentation was attributed to demand-related ischemia. The patient was stabilized with blood transfusions and cessation of clopidogrel. He remained neurologically intact throughout. The chest pain and ECG changes resolved as Hb improved (Figure 1).

An inpatient colonoscopy revealed incidental colonic polyps but no evidence of active bleeding. On the advice of the gastroenterology team, a CT enteroclysis was performed; however, the results were inconclusive, and the cause of the GI bleed remained unidentified.

**Figure 2:**
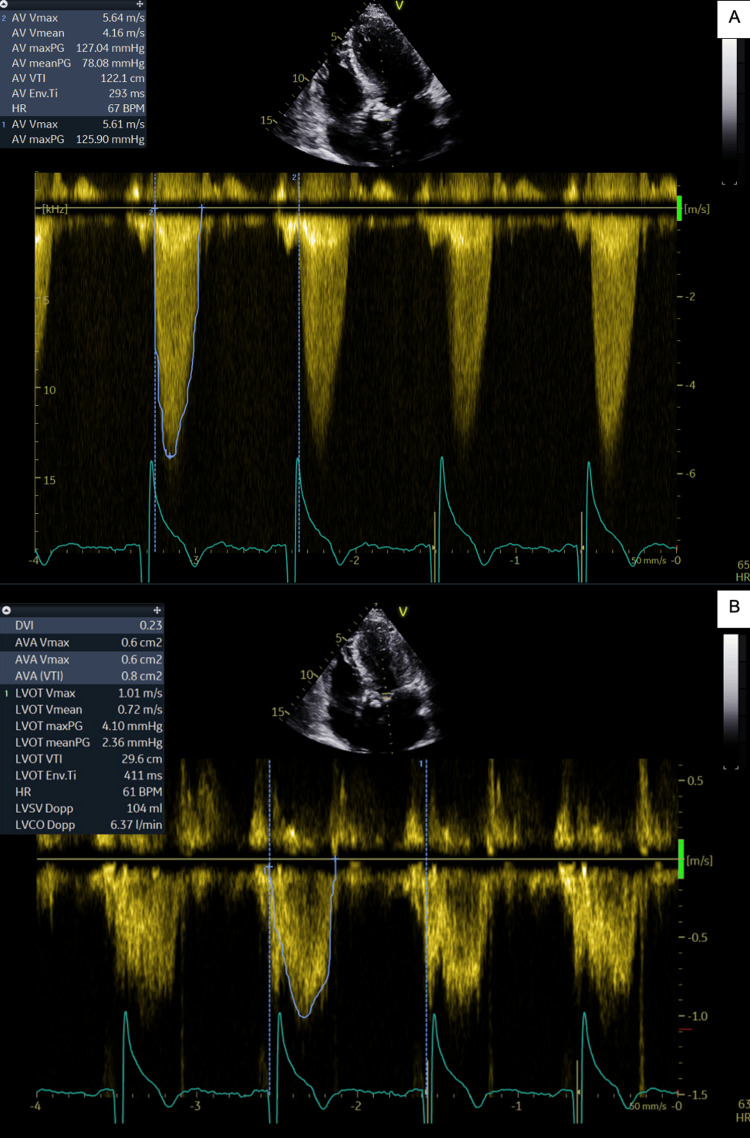
Pre-TAVI echocardiogram showing signs of severe AS (A) Continuous-wave Doppler demonstrating severe AS with an aortic valve Vmax of 5.67 m/s (severe >4 m/s). (B) Pulsed-wave Doppler of the left ventricular outflow tract, used in the continuity equation to calculate a valve area of 0.6 cm² (severe <1 cm²) [6]. AS, aortic stenosis; TAVI, transcatheter aortic valve implantation

During the index admission, echocardiography revealed very severe AS (Vmax >5 m/s) (Figure 2). Before the scheduled follow-up or colonoscopy could take place, the patient was readmitted with symptomatic anemia, with Hb measuring 68 g/L. Two additional units of blood were transfused, leading to stabilization of both Hb and symptoms. He was subsequently discharged with a plan for an outpatient colonoscopy.

**Figure 3:**
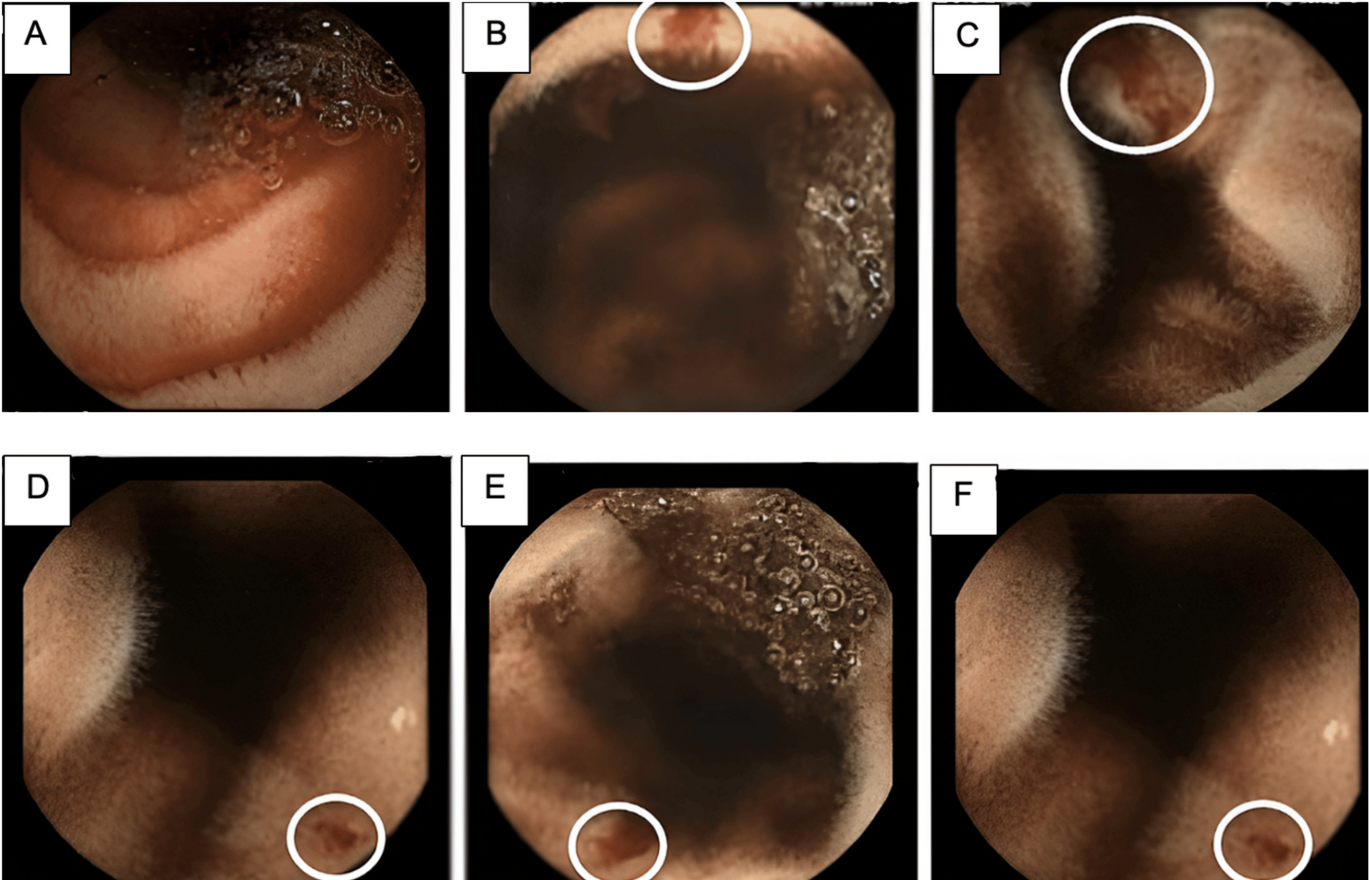
Capsule endoscopy images of the small bowel (A) Luminal blood in the jejunum. (B-F) Areas of bleeding angioectasia in the jejunum and ileum (circled in white).

At this stage, the patient continued to experience GIB and required a total of 13 units, nearly 6 L, of blood to maintain Hb levels and prevent ischemic symptoms. A diagnosis of Heyde’s syndrome was suspected, characterized by angiodysplasia in the setting of severe AS. Following continued melena and consultation with the gastroenterology team regarding the potential diagnosis, a capsule endoscopy (PillCam™, Medtronic plc, Minneapolis, MN, USA) was arranged. Similar to colonoscopy, bowel preparation for capsule endoscopy involves dietary restrictions, including a low-fiber diet followed by a clear liquid diet starting the day before the procedure, along with a laxative solution. The images, interpreted by the local gastroenterology team, confirmed actively bleeding angioectasia in the jejunum and ileum (Figure 3).

Conventional treatments for small bowel UGIB, such as double-balloon endoscopy, were considered; however, given the context of Heyde’s syndrome, TAVI was deemed the first-line treatment.

**Figure 4:**
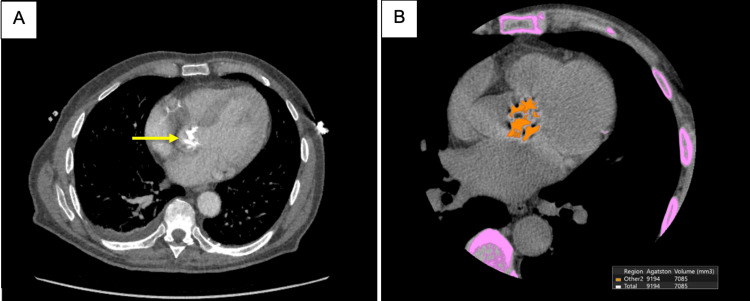
(A) CT coronary angiogram showing a calcified aortic valve (yellow arrow). (B) Calcium scoring demonstrating aortic valve calcification with an Agatston score of 9,194 highlighted in orange. The yellow arrow indicates the calcified aortic valve, while the orange area represents the aortic valve with calcium scoring software applied to calculate the Agatston score. Purple areas represent bone, used as a reference to quantify the degree of calcification.

A CT coronary angiogram was performed prior to the planned TAVI, demonstrating an aortic valve Agatston score of 9,194 units (Figure 4). No obstructive coronary artery disease was identified on invasive coronary angiography.

**Figure 5:**
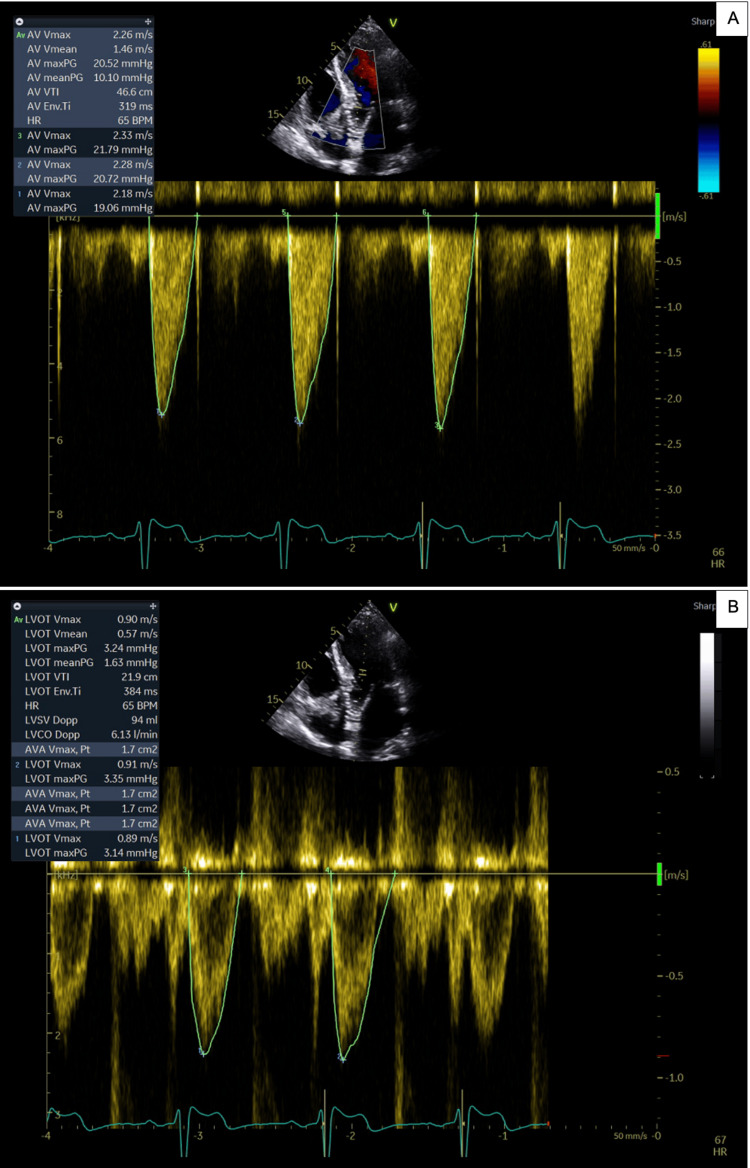
Post-TAVI echocardiogram demonstrating a well-seated valve (A) Continuous-wave Doppler showing normal aortic Vmax (normal <2.5 m/s). (B) Pulsed-wave Doppler demonstrating an aortic valve area of 1.7 cm² [6]. TAVI, transcatheter aortic valve implantation

The patient was transferred to the local cardiothoracic surgical center, where he underwent a successful TAVI procedure using a self-expandable Evolut FX valve. Post-procedure echocardiography confirmed that the valve was well seated, with minimal regurgitation and good overall function (Figure 5). Three additional units, approximately 1.5 L, of blood were required in the post-TAVI period due to bleeding from femoral access sites and residual GI angioectasia. The patient was subsequently monitored by the gastroenterology team. His Hb stabilized, and he was discharged in good condition. He has since remained well and was able to restart his antiplatelet therapy without further bleeding. As an outpatient, he underwent peroral double-balloon enteroscopy to evaluate the small bowel directly, which showed resolution of the angiodysplasia.

As the presenting ECG (Figure 1) showed widespread ST depression consistent with ischemia, it was essential that this was promptly investigated. Treatment is guided by the most likely underlying diagnosis. In cases where acute coronary syndrome (ACS) is suspected, percutaneous coronary intervention is the treatment of choice. However, in cases such as this, where type 2 myocardial infarction secondary to demand-related ischemia is suspected, further investigations and management should focus on treating the underlying cause.

The timeline of symptoms is a useful guide in distinguishing the cause of myocardial ischemia. In type 1 ischemia, caused by ACS, the initial symptoms typically include chest pain and autonomic manifestations [7]. In this case, melena with significant anemia preceded the onset of these symptoms, suggesting a type 2 ischemic pattern. Differentiating between ACS and demand-related ischemia is particularly important, as treatment for the former involves antithrombotic therapy. The patient underwent coronary angiography, which did not reveal any coronary artery disease that could account for the presentation. In this context, the bleeding angioectasia, already causing substantial end-organ hypoperfusion, as evidenced by the troponin elevation and ECG findings, could have led to further deterioration and potentially fatal outcomes.

To effectively treat the bleeding source, its location needed to be identified. However, the various endoscopic investigations, CT scans, and ultimately capsule endoscopy required considerable time to complete. Capsule endoscopy is not a first-line investigation for GIB, and as a result, the time to diagnosis and treatment in cases of Heyde’s syndrome can be prolonged. This condition is often associated with quasi-occult, low-volume small bowel bleeding [5], leading to extended hospital stays and the need for substantial supportive therapy. In this case, a total of 13 blood transfusions and an intravenous iron infusion were required before definitive treatment with TAVI.

In Heyde’s syndrome, surgical AVR (SAVR) has previously been shown to address all three components of the triad [4]. In this case, considering the patient’s age and comorbidities, valve replacement was performed using a minimally invasive, endovascular approach with TAVI. The patient experienced an uneventful postoperative course and was discharged with a stable Hb level. He has since remained well. This case demonstrates the efficacy of TAVI as a definitive treatment for Heyde’s syndrome.

Serial echocardiograms were used to monitor the patient’s aortic valve following TAVI (Table 1). As shown, both visual and Doppler assessments indicated marked improvement in valve function. The aortic valve area increased from 0.6 cm² to 1.7 cm², and the maximum velocity across the valve decreased from 5.67 m/s to 2.26 m/s. Normalization of aortic valve function (Table 1) coincided with stabilization and subsequent normalization of Hb levels, as well as resolution of small bowel angiodysplasia. The double-balloon enteroscopy performed during follow-up confirmed complete resolution of the angiodysplasia, supporting the conclusion that TAVI can serve as an effective and definitive treatment for Heyde’s syndrome.

**Table 1:** AS severity grading AS, aortic stenosis Adapted from Heyde (1958) [6]

Grade	Mild	Moderate	Severe
Peak velocity (m/s)	2.5-2.9	3.0-3.9	>4.0
Mean gradient (mmHg)	<20	20-39	>40
Valve area (cm²)	>1.5	1.0-1.5	<1.0

## Discussion

UGIB is a common presentation to emergency departments and may manifest as hematemesis, melena, or a combination of both in over one-third of cases [5]. Clinicians evaluating these patients must carefully assess both medical and social histories to identify the most likely diagnosis and initiate appropriate investigations and management [5].

**Figure 6:**
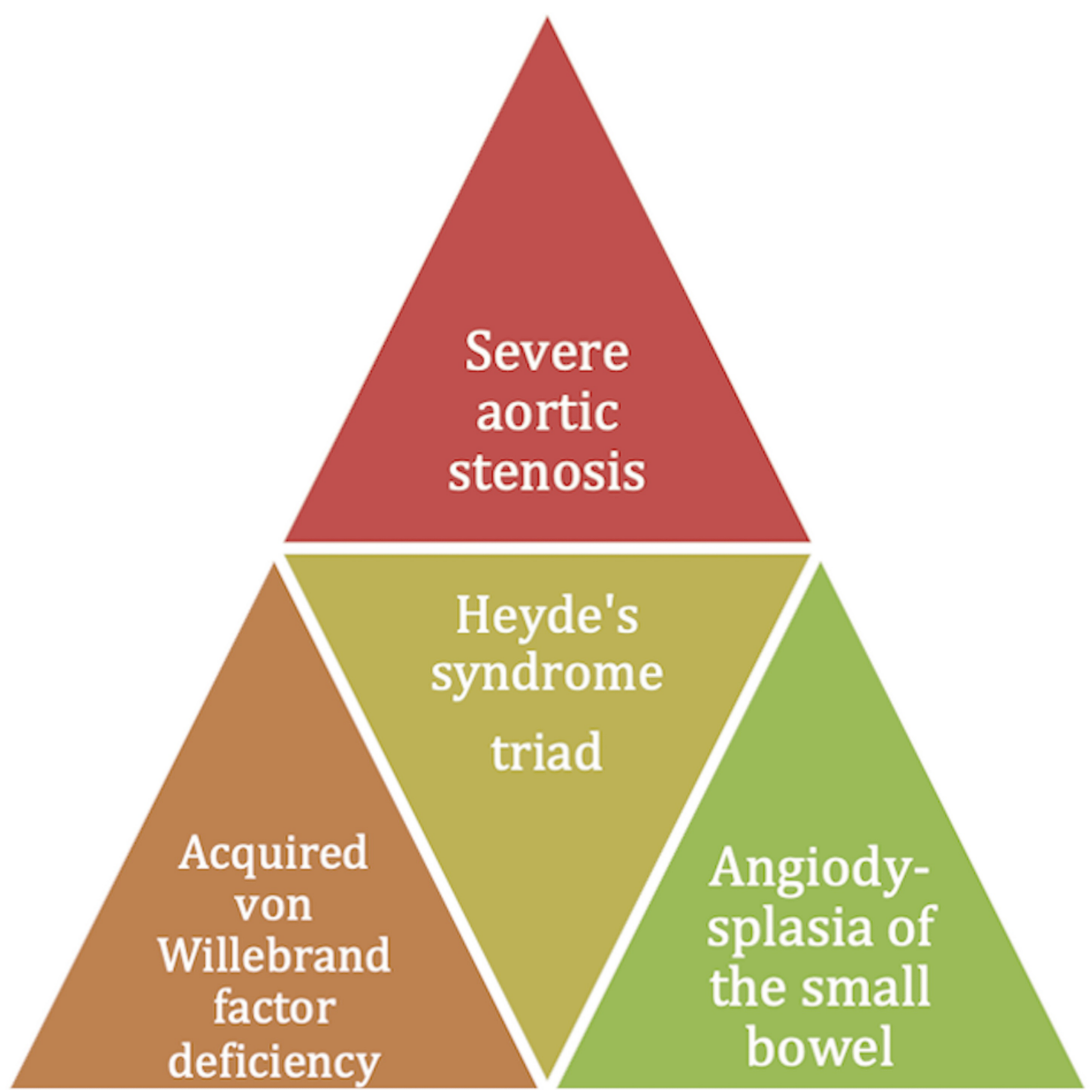
Heyde’s syndrome triad Adapted from Saha et al. (2022) [7]

A less frequent cause of GIB is angiodysplasia associated with severe AS. This condition, together with AVWS-2A, forms the syndromic triad known as Heyde’s syndrome, as illustrated in Figure 6 [7]. AS affects approximately 7.5% of individuals over the age of 75, with 1.8% falling into the moderate or severe categories [3]. Although data on the prevalence of Heyde’s syndrome within this population are limited, significant GIB has been reported in 1-3% of cases [4]. Moreover, studies have shown that even in the absence of clinically significant bleeding, up to 70% of patients with AS exhibit a reduction in VWF, which improves following successful surgical valve replacement [8].

Initial management principles for UGIB include resuscitation, correction of coagulopathy, and stabilization of hemodynamic status. The timing of endoscopy depends on the clinical course and suspected underlying etiology. If a history of severe AS has not previously been established by echocardiography, diagnosis may rely on clinical examination. The classical finding in severe AS is an ejection systolic murmur radiating to the carotids, accompanied by a slow-rising pulse and a diminished or absent second heart sound [3].

The triad of AS, UGIB, and AVWS, known as Heyde’s syndrome, is most often associated with bleeding from small bowel angiodysplasia. Because bleeding typically occurs distal to the duodenum, initial OGD, followed by colonoscopy and CT imaging, often fails to identify the bleeding source [5]. Awareness of Heyde’s syndrome is crucial, as early video capsule endoscopy can expedite identification of the culprit lesion [7]. As demonstrated in this case, delays in diagnosis can result in significant GIB, profound anemia, and ischemic complications due to reduced oxygen delivery. Importantly, patients with AS are more likely to have underlying coronary artery disease, making them less tolerant of severe anemia [9].

With an aging population, the prevalence of AS and its associated health and economic burden is expected to rise [10]. Consequently, awareness of AS-related complications such as Heyde’s syndrome will become increasingly important. In this case, bleeding resolved following correction of the severe AS through TAVI. With over 3,000 TAVI procedures performed annually in the UK, this treatment option is becoming increasingly accessible across the country [10].

**Figure 7:**
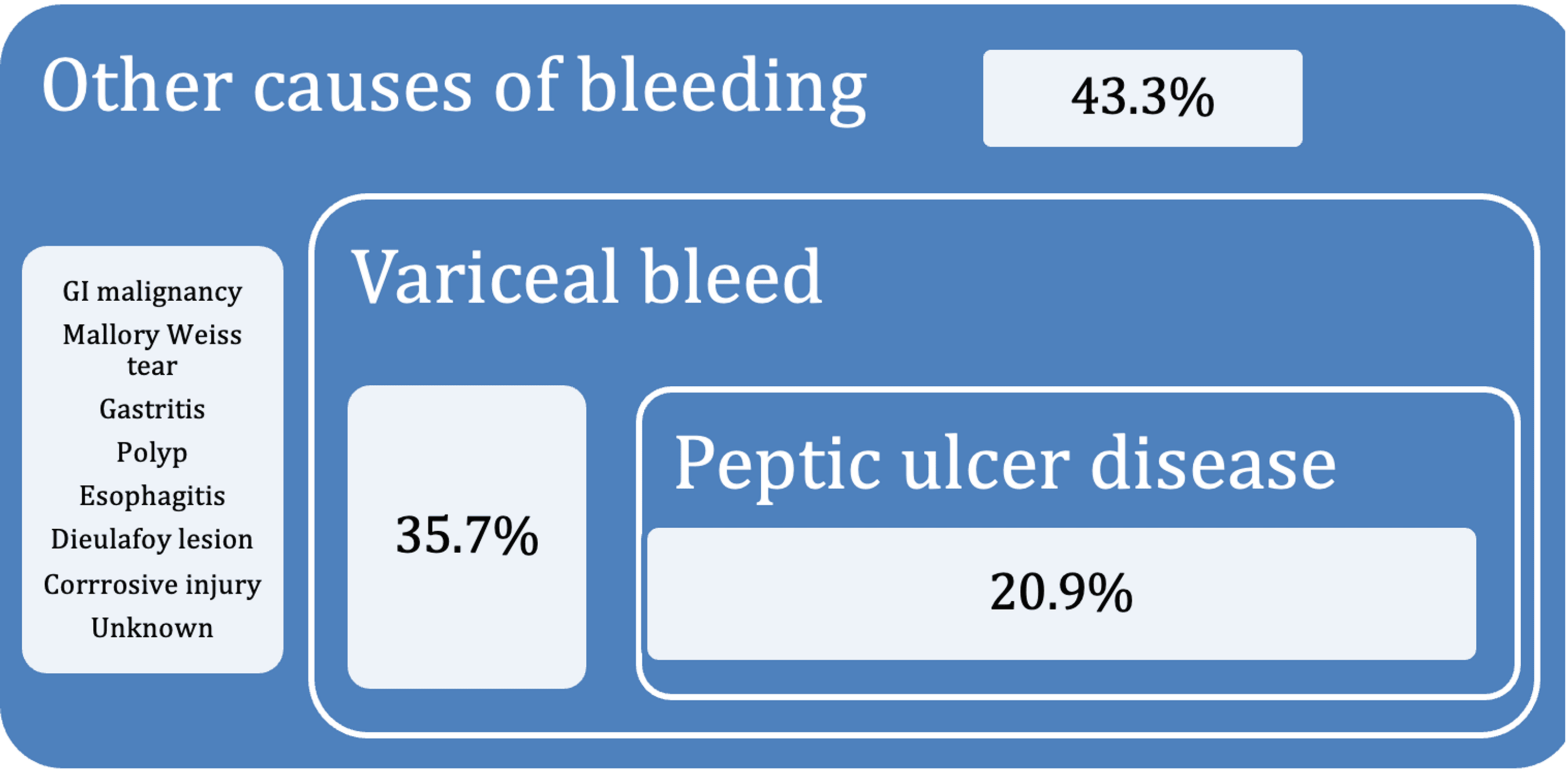
Causes of UGIB UGIB, upper GI bleeding Adapted from Shenoy et al. (2021) [5]

The location of GIB can often be inferred from the nature of the presenting symptom. Dark melena typically indicates UGIB (from the stomach or small bowel proximal to the ligament of Treitz), whereas fresh red bleeding suggests lower GIB (from the large bowel) or a brisk upper GI bleed that has not been digested [5]. In this case, the patient presented with melena, prompting an initial OGD to identify common causes of UGIB. Figure 7 illustrates the incidence of confirmed etiologies of UGIB based on data from Shenoy et al., derived from 74,466 emergency department presentations over a six-month period. In addition to bedside and endoscopic evaluations, the diagnosis of each cause depends heavily on the patient’s medical history [5].

**Figure 8:**
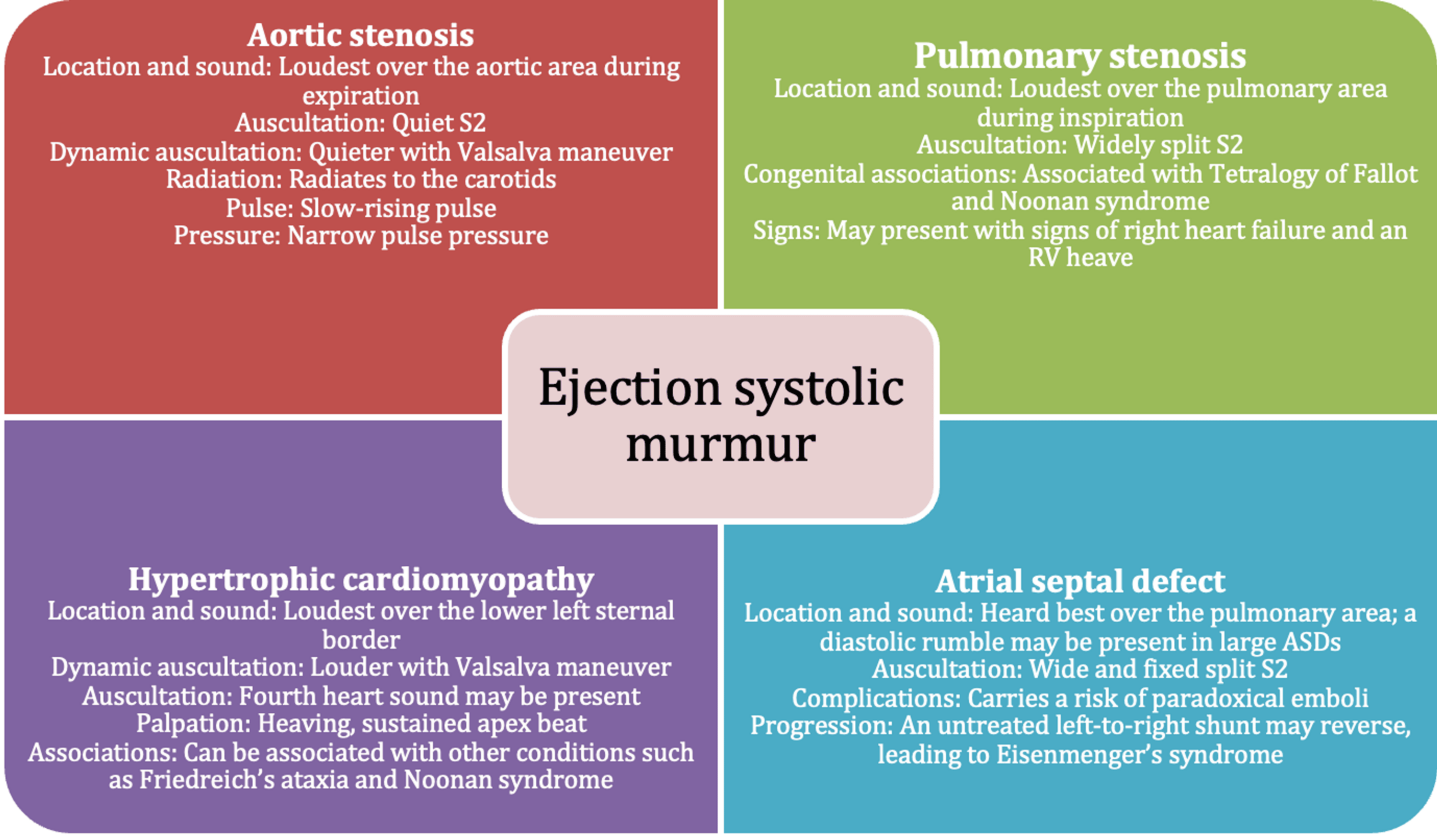
Causes of an ejection systolic murmur ASD, atrial septal defect; RV, right ventricular Adapted from Alpert (1990) [11]

When evaluating patients for AS, the diagnosis is confirmed by echocardiography. Prior to imaging, it may be suspected that an ejection systolic murmur is auscultated that radiates to the carotid arteries. Other clinical signs may include a slow-rising pulse, a narrow pulse pressure, and a quiet S2 [11]. Figure 8 outlines other causes of ejection systolic murmurs and how these may be differentiated on clinical examination.

A diagnosis of Heyde’s syndrome should be suspected in patients with GIB and AS, as in this case. However, it is also important to exclude more common causes of GIB through upper and lower GI endoscopy before proceeding to video capsule endoscopy. Upper GI endoscopy is particularly important to perform early due to the potential for endoscopic treatment in cases such as variceal or peptic ulcer disease. Once upper and lower GI endoscopies have excluded common causes, clinicians should proceed to assess the small bowel with investigations such as small bowel capsule endoscopy to ensure a timely diagnosis in suspected Heyde’s syndrome.

The pathophysiology of Heyde’s syndrome is thought to center around the reduction in functional VWF, a protein essential for platelet adhesion and hemostasis [12]. The high shearing forces exerted on circulating VWF proteins as they cross the stenotic aortic valve lead to conformational changes and proteolytic degradation [12]. The resulting acquired VWF deficiency (AVWS-2) ultimately causes defective platelet adhesion and a bleeding diathesis [12]. As seen in our case, this often manifests clinically as bleeding from GI angiodysplasia.

A 2023 systematic review and meta-analysis by Goltstein et al. assessed the treatment of Heyde’s syndrome with AVR, comparing SAVR and TAVI. Forty-four observational studies were included: 11 evaluated GIB outcomes (n = 300), 32 assessed AVWS (n = 1,054), and one reported on both conditions (n = 6). The analysis showed that following successful valve replacement, recovery of AVWS and cessation of GIB occurred in 87% and 73% of cases, respectively. The overall complete cessation of GIB was lower in the TAVI group (64%) compared with the SAVR group (82%). The main factor associated with rebleeding was residual valve disease [13]. The authors hypothesized that the efficacy of TAVI in this context will likely improve as technology and operator experience advance.

A 2013 retrospective study of 400 patients by Godino et al. found that up to 6% of patients undergoing TAVI had small bowel angiodysplasia, with 1.7% diagnosed with Heyde’s syndrome. Similar to the patient presented in our case, post-procedure GIB from angiodysplasia occurred, requiring more than three units of blood transfusion. Interestingly, all patients who were successfully treated with TAVI had no further rebleeding at follow-up (22 ± 15 months). Ongoing GIB requiring transfusion occurred in only one Heyde’s syndrome patient in whom TAVI had failed [14].

TAVI is a minimally invasive procedure that avoids the need for cardiopulmonary bypass and sternotomy, with the added benefit of being performed under local anesthesia [15]. This eliminates the need for general anesthesia and its associated risks. Although TAVI has traditionally been reserved for patients at high surgical risk, recent research demonstrates its potential efficacy in lower-risk groups [15]. A 2025 systematic review that included six studies and 5,341 lower-risk patients (2,717 randomized to TAVR and 2,624 to SAVR; weighted mean follow-up of 35.7 months) found that at five years, TAVR was associated with a 20% reduction in the hazard of all-cause death (HR: 0.80; 95% CI: 0.66-0.97; P = 0.02) and a 19% reduction in the hazard of all-cause death or disabling stroke (HR: 0.81; 95% CI: 0.68-0.96; P = 0.01) compared with SAVR [15].

The NHS position statement also notes that TAVI is associated with a shorter hospital stay, three days for TAVI compared with seven days for SAVR, demonstrating an additional benefit in terms of resource utilization [16]. Table 2 summarizes the inclusion and exclusion criteria from the NHS position statement.

**Table 2:** NHS position statement on inclusion and exclusion criteria for TAVI ACC, American College of Cardiology; AHA, American Heart Association; AS, aortic stenosis; EACTS, European Association for Cardio-Thoracic Surgery; ESC, European Society of Cardiology; MDT, multidisciplinary team; NYHA, New York Heart Association; TAVI, transcatheter aortic valve implantation Adapted from [16]

Eligibility criteria	Exclusion criteria
Patients must have severe AS confirmed by a multidisciplinary team and in accordance with ESC/EACTS and ACC/AHA guidelines.	When comorbidity, frailty, or limited life expectancy (less than one year) makes the intervention inappropriate
Patients must be symptomatic, presenting with one or more of the following: NYHA class > I, chest pain, shortness of breath, presyncope, or syncope.	Patients who are asymptomatic despite severe AS
Patients must be reviewed and discussed at an aortic valve MDT meeting.	Individuals deemed unsuitable for TAVI following assessment at an aortic valve MDT meeting

## Conclusions

This case underscores the diagnostic challenge of Heyde’s syndrome in a patient presenting with recurrent GIB and severe AS, where initial upper and lower GI endoscopies were negative. The key learning point is the importance of considering this diagnosis early, which should prompt investigation of the small bowel. Video capsule endoscopy is often required to identify the characteristic angioectasia, as the bleeding source is frequently inaccessible with standard endoscopy. Clinicians should also recognize that an acquired von Willebrand deficiency is common in patients with significant AS, contributing to a bleeding tendency even before angioectasia becomes clinically apparent.

The definitive management hinges on treating the underlying valvular lesion rather than the secondary GI lesions. This case demonstrates that AVR, including via the minimally invasive TAVI procedure, is the cornerstone of treatment, effectively reversing the hemodynamic drivers of the coagulopathy. While surgical replacement has historically been the mainstay, TAVI represents a viable alternative, particularly for higher-risk patients. The critical factor for a successful outcome, regardless of approach, is the implantation of a well-seated valve that eliminates the pathological shear stress responsible for the syndrome. This highlights the paramount importance of a multidisciplinary strategy between cardiology and gastroenterology to guide appropriate diagnosis, intervention, and monitoring. As TAVI becomes increasingly accessible across the UK, its role in the effective management of this complex syndrome is expected to expand.

## Appendices

Narrative from the patient’s perspective

My experience leading up to my heart operation (TAVI) was really frightening. Being told I was bleeding internally was daunting, and going in and out of the hospital for transfusion after transfusion, along with undergoing various tests, was very stressful. Once I had the heart operation (TAVI), I seemed to turn a corner. I am now well; all tests show no bleeding, and my heart is functioning properly. Cardiology continues to monitor me closely. I am very grateful to all the nurses and doctors who cared for me across all three hospitals.

## References

[1] Ring L, Shah BN, Bhattacharyya S (2021). Echocardiographic assessment of aortic stenosis: a practical guideline from the British Society of Echocardiography. Echo Res Pract.

[2] Ojha N, Dhamoon AS (2025). Myocardial infarction. StatPearls [Internet].

[3] Lourdusamy D, Mupparaju VK, Sharif NF, Ibebuogu UN (2021). Aortic stenosis and Heyde's syndrome: a comprehensive review. World J Clin Cases.

[4] Vincentelli A, Susen S, Le Tourneau T (2003). Acquired von Willebrand syndrome in aortic stenosis. N Engl J Med.

[5] Shenoy V, Shah S, Kumar S (2021). A prospective cohort study of patients presenting to the emergency department with upper gastrointestinal bleeding. J Family Med Prim Care.

[6] Heyde E (1958). Gastrointestinal bleeding in aortic stenosis. New Eng J Med.

[7] Saha B, Wien E, Fancher N, Kahili-Heede M, Enriquez N, Velasco-Hughes A (2022). Heyde's syndrome: a systematic review of case reports. BMJ Open Gastroenterol.

[8] Anderson R (1996). Reversal of aortic stenosis, bleeding gastrointestinal angiodysplasia, and von Willebrand syndrome by aortic valve replacement. Lancet.

[9] de Azevedo Filho AF, Accorsi TA, Ribeiro HB (2021). Coronary artery disease in patients with aortic stenosis and transcatheter aortic valve implantation: implications for management. Eur Cardiol.

[10] Ludman PF (2019). UK TAVI registry. Heart.

[11] Alpert MA (1990). Systolic murmurs. Clinical Methods: The History, Physical, and Laboratory Examinations. 3rd edition.

[12] Maksić M, Corović I, Stanisavljević I (2024). Heyde syndrome unveiled: a case report with current literature review and molecular insights. Int J Mol Sci.

[13] Goltstein LC, Rooijakkers MJ, Hoeks M (2023). Effectiveness of aortic valve replacement in Heyde syndrome: a meta-analysis. Eur Heart J.

[14] Godino C, Lauretta L, Pavon AG (2013). Heyde's syndrome incidence and outcome in patients undergoing transcatheter aortic valve implantation. J Am Coll Cardiol.

[15] Reddy RK, Howard JP, Mack MJ (2025). Transcatheter vs surgical aortic valve replacement in lower-risk patients: an updated meta-analysis of randomized controlled trials. J Am Coll Cardiol.

[16] (2025). Transcatheter aortic valve implantation (TAVI) and surgical aortic valve replacement (SAVR) for symptomatic, severe aortic stenosis (adults) to support elective performance. https://www.england.nhs.uk/long-read/tavi-and-savr-position-statement/.

